# Association between Depressive Symptoms, Physical Activity, and Health Factors in Hispanic Emerging Adults

**DOI:** 10.3390/ijerph21070918

**Published:** 2024-07-14

**Authors:** Margaret Gutierrez, Cristina Palacios, Vijaya Narayanan, Florence George, Sabrina Sales Martinez

**Affiliations:** 1 Robert Stempel College of Public Health and Social Work, Florida International University, Miami, FL 33199, USA; mguti140@fiu.edu (M.G.); crpalaci@fiu.edu (C.P.); vnarayan@fiu.edu (V.N.); 2 College of Arts, Sciences and Education, Florida International University, Miami, FL 33199, USA; fgeorge@fiu.edu

**Keywords:** depression, physical activity, modifiable lifestyle factors, stress, emerging adults, body composition

## Abstract

Physical activity is a modifiable lifestyle behavior known for reducing symptoms of and being a risk factor for depression and mental health disorders. However, emerging adults (ages 18–25) struggle to meet recommended amounts. In this study, we explore the association between physical activity, depressive symptoms, and health factors in 137 Hispanic emerging adults. Using a cross-sectional survey design, sociodemographic information, depressive symptoms (CES-D score), physical activity (IPAQ score), body composition, and blood pressure measures were obtained. Statistical analyses included correlation and regression analyses. More than half of the participants demonstrated depressive symptomology (59.1%) and body fat percentage greater than 25% (64.2%). Body fat percentage, lean body mass, stress, and heart rate demonstrated notable associations with depressive symptoms and physical activity. When measured continuously and categorically, IPAQ was not a significant predictor of depressive symptoms. When used as a binary variable with a cutoff of 600 MET min/week, IPAQ score revealed a negative relationship with CES-D score (β = −0.169, SE = 2.748, *p* = 0.034). Our results indicate that a threshold of physical activity, 600 MET min/week, may confer protective effects against depressive symptoms. Future research should investigate the context and quality of physical activity to address mental health disparities in this underrepresented population.

## 1. Introduction

The transition into adulthood, from ages 18 to 25 years, is known as emerging adulthood and is characterized by several notable changes. Emerging adults are navigating employment and/or higher education, while simultaneously trying to establish social networks and lifestyle behaviors independent from their families. This may explain the high prevalence of mental health disorders in this age group and why symptoms typically manifest during this period [1,2,3]. Physical activity is widely recognized as a modifiable risk factor and adjunctive or alternative treatment for depression and other mental health disorders [4,5,6]. However, emerging adults struggle to meet recommended activity levels due to their dynamic and evolving lifestyles. 

Depression is a complex mental health disorder with symptoms such as low mood and motivation, dysphoria, and other impairments [7]. Physical activity addresses the multifaceted symptoms suffered in those with depression through several mechanisms. Along with decreasing levels of pro-inflammatory mediators and reactive oxygen species, exercise is associated with improved cerebral blood flow and stress response [7,8,9]. Physical activity also influences psychosocial outcomes by improving self-esteem, increasing self-efficacy, and mitigating the effects of adverse childhood experiences [9,10]. As little as 10–30 min of exercise can lead to enhancements in mood and various modalities have proven to be effective at reducing depressive symptoms [11].

The United States (U.S.) Department of Health and Human Services and the World Health Organization recommend at least 150 min per week of moderate-intensity physical activity that incorporates aerobic exercise, resistance training, flexibility, and balance components for adults [12,13]. Both aerobic and resistance training have positive impacts on mood, attention, memory, and cognitive development [14,15,16]. Recent systematic reviews have indicated several modalities are effective alternatives or adjuncts to selective serotonin reuptake inhibitors for depressive symptoms when at least 150 min per week of varying intensities are met for at least 3 weeks [4,5]. Together they also help maintain lean body mass and functional mobility, regulate hormones and metabolic rate, and enhance vascular function [17,18,19,20]. In addition to the well-established cardiovascular benefits of aerobic activity, recent data highlights the positive impact of resistance training on heart rate and blood pressure measures [21,22,23]. 

Research has consistently shown a concerning decline in physical activity among adolescents, a trend that continues to worsen in young adulthood while juggling newfound independence and responsibilities [24,25]. Physical inactivity and sedentarism contribute to progressive loss of skeletal muscle and obesity associated with the risk of suffering from depression and cardiometabolic multimorbidity with age [26,27]. From 2008 to 2016, less than 30% of adolescents in the U.S. reported sufficient levels of physical activity [12]. This is especially concerning considering the significant decline in activity with transition into adulthood and age. In 2020, only 24.2% of adults met the guidelines for both aerobic and resistance training activities [28]. In adults 18–34, only 41.3% of men and 22.7% of women met both physical activity guidelines [28]. 

This decline in physical activity is especially pronounced in Hispanics, who report less physical activity with years lived in the U.S. [29,30,31]. Hispanics are also particularly vulnerable to depression and other mental health disorders [32,33,34,35,36]. Several factors such as socioeconomic disparities, limited access to recreational facilities in neighborhood environments, adverse childhood experiences, and the stress of acculturation may contribute to these trends [32,33,34,35,36]. According to the Hispanic Community Health Study/Study of Latinos (HCHS/SOL), Hispanic adolescents only perform 35 min of moderate to vigorous physical activity per day despite a recommendation of at least 60 min per day in those under 18 years old [12,29,37]. Between 2015 to 2020, Hispanic adults reported the highest prevalence of physical inactivity compared to non-Hispanic Black and White adults [38,39]. However, limited information is available regarding physical activity in Hispanic emerging adults as much of the current data focuses on children and older adults. Emerging adulthood is a transitional period characterized by increased autonomy and the development of lifestyle habits that can have lasting effects on health. Therefore, it is a critical time to address potential disparities and understand how they may influence the risk of suffering from depression and multimorbidity later in life. The purpose of this study was to explore the association between depressive symptoms, physical activity, and modifiable lifestyle factors among Hispanic emerging adults. 

## 2. Materials and Methods

### 2.1. Design and Recruitment

A cross-sectional design was used to examine the association between depressive symptoms, physical activity, and other health factors in Hispanic emerging adults (18–25 years). Study procedures were approved by the Florida International University (FIU) International Review Board prior to recruitment. Participants were recruited using convenience sampling if they were 18 to 25 years old, self-identified as Hispanic, and willing to share their data. They were excluded if they were outside of the age range, not Hispanic, or had low literacy which prevented them from completing the questionnaire. Recruitment and data collection took place from August 2022 through April 2023, and a total of 196 participants were recruited primarily from a university setting. Study flyers were distributed around campus and emailed to professors for them to share with students. 

### 2.2. Procedures

Participants attended an initial visit where a screening checklist was completed to ensure eligibility and literacy. Informed consent was signed after a thorough explanation of study procedures. Participants were sent links by email or text to complete the survey online. After completing the survey, participants attended a visit where blood pressure and body composition measures were taken. Once all measures were obtained, participants received an incentive in the form of a USD 10 gift card. 

### 2.3. Assessments

#### 2.3.1. Survey/Questionnaire

An online survey was used to collect sociodemographic information, self-reported chronic conditions, and information about depressive symptoms, stress, and physical activity, using validated instruments as described below. 

Sociodemographic information: The survey gathered age, sex, race, time in the U.S., education, major at FIU, income level, employment status, perceived feeling of safety at home, health insurance, household size, and self-reported chronic conditions, among others. Participants were asked the question “How stressed do you currently feel?” to rate their stress using a Likert scale (1. Not at all, 2. A bit, 3. Something, 4. Enough, 5. A lot). They were also asked to identify their main cause of stress, including options such as finances, familial or health issues, work-related concerns, and worldly events amongst others. 

Depressive symptoms: The main outcome variable was measured using the Center for Epidemiological Studies Depression (CES-D) Survey, a 20-item widely used screening tool assessing feelings experienced in the last week such as “I had trouble keeping my mind on what I was doing” and “I felt everything I did was an effort.” Respondents answered on a four-point scale: “Rarely or none of the time (<1 day)”, “Some or a little of the time (1–2 days)”, “Occasionally or a moderate amount of the time (3–4 days)”, “Most or all of the time (5–7 days)” [40,41]. A total score of 16 or more is indicative of clinically significant depressive symptomology [42]. Radloff et al. confirmed its reliability and validity in adolescents and young adults [41]. It has been translated to and validated for use in Spanish and several other languages [43,44]. 

Physical activity: Measured using the International Physical Activity Questionnaire (IPAQ), a commonly used tool to comprehensively measure activity from four possible domains: during transportation, at work, during household and gardening tasks, and during leisure time, including exercise and sports, with varying intensities—moderate, vigorous, and walking [45,46]. The long form used in our survey includes 27 items where participants enter how many minutes they spent in each activity during the last week. This was then used to calculate a total IPAQ score as Metabolic Equivalent Task (MET) minutes per week. 

The IPAQ score provides a MET min/week score for each intensity—vigorous physical activity (VPA), moderate physical activity (MPA), and walking—and domain—at work, during active transport, during housework, and during leisure time. A MET can be calculated as 3.5 milliliters of oxygen consumed per kilogram of body weight per minute while performing an activity; one MET is the energy typically expended while sitting or at rest [12]. Moderate-intensity activities are defined as 3.0–5.9 METs, while vigorous-intensity activities are categorized as 6.0 METs or more. The recommendation of 150 to 300 min of moderate-intensity physical activity or 75 to 150 min of vigorous-intensity physical activity is equivalent to about 500–1000 MET minutes per week [12]. 

Responses from the survey were scored using the Guidelines for Data Processing and Analysis of the IPAQ [47]. A spreadsheet was created to allow for manual entry of participant survey responses and calculation using embedded formulas. MET values were calculated for each domain using the formulas provided within the scoring guideline by multiplying an activity factor for each intensity by the number of minutes and days spent in that activity. The total MET minutes per week were calculated as the sum of walking, moderate, and vigorous MET-min/week scores at work, during transportation, during household and gardening tasks, and during leisure time. Physical activity levels were categorized as low (<600 METs/week), moderate (600–1500 METs/week), and high (>1500 METs/week) to be used as an ordinal categorical variable [48]. Physical activity was also assessed as a binary variable, greater than or equal to 600 METs/week indicating an adequate level of activity [12,13]. 

#### 2.3.2. Blood Pressure

Three blood pressure measurements were taken 60 s apart using a digital upper arm blood pressure monitor while the participant was seated at rest (Welch Allyn ProBP 2400 Digital Blood Pressure Device, Microlife Corporation, Taipei, Taiwan). Blood pressure measurements were taken in the right arm unless participant reported any reason that the measurement should not be taken in the right arm. The digital monitor used simultaneously measured heart rate during blood pressure measurements. The average of the three measures was reported. 

#### 2.3.3. Anthropometrics and Body Composition

Height was measured using a stadiometer (Detecto, Fisher Weighing Systems Inc., Webb City, MO, USA). Weight and body composition measures including lean body mass (LBM), total body water, and body fat percentage were collected using a bioelectrical impedance machine (InBody 520™, Biospace, Los Angeles, CA, USA) that also calculated body mass index (BMI) (kg/m^2^) and basal metabolic rate (BMR) [49]. The InBody 520™ uses eight polar tactile electrodes to send varying frequencies through the body to differentiate between fat and fat-free mass, with a 98% correlation to Dual-Energy X-ray Absorptiometry [50,51,52]. Participants were asked to remove jewelry, excessive clothing, and shoes before examination. Participants were then instructed to wipe their palms and soles of their feet with an InBody tissue to enhance the electrical conductivity between hands, feet, and electrodes. Waist circumference was measured with a tape measure at the midpoint between the lower rib and the iliac crest in centimeters. Hip circumference was measured at the widest part of the hips, also in centimeters. 

### 2.4. Data Analysis

We showed descriptively the mean and standard deviation for continuous variables and the frequency and percent for categorical values. We used independent-sample *t*-tests to compare continuous variables between individuals with CES-D scores less than 16 or ≥16, indicating depressive symptomology [42], and for those with IPAQ scores less than or ≥600 MET min/week, which aligns with physical activity recommendations [12]. Chi-square was employed for categorical variables. Results from Fisher’s exact test were reported if the expected frequency of one or more cells was less than 5. Spearman’s correlation was used to measure the magnitude of association between depressive symptoms, physical activity, and other variables. Linear regression was run to understand the relationship between physical activity and CES-D score. The first regression model included the total IPAQ score, while the following models assessed IPAQ as ordinal categories (0–600 MET min/week, 600–1499 MET min/week, and ≥1500) and binary scores (0–599 MET min/week and ≥600) respectively. Linear regression was controlled for sex, race, education level, body fat percentage, stress, and being a psychology major, as these factors are hypothesized to influence mental health outcomes [53,54,55]. The goal of the logistic regression analysis was to examine if the IPAQ score cutoff point of 600 MET min/week, which indicates adequate physical activity, predicted a lesser likelihood of depressive symptoms, as indicated by CES-D score >16. In the linear regression, Model 1 included the total IPAQ score, while the following models assessed IPAQ categorically, dichotomously, and as varying intensities and domains, respectively. Logistic regression was controlled for other variables that may be associated with other predictors and dependent variables, which included sex, race, country of origin, time in the U.S., primary language, education level, employment, stress, body fat percentage, and being a psychology major. These variables were included in the regression models because several studies have documented their influence on both mental health outcomes and modifiable behaviors, especially in Hispanic populations [53,54,55]. Significance was set at an alpha value of 0.05 for analyses. SAS 9.4 and SPSS 26 and 28 were used for data analysis.

## 3. Results

### 3.1. Health-Related and Demographic Characteristics by CES-D and IPAQ Scores

Out of the 140 participants who completed all the steps of the study protocol, 3 were excluded due to missing data. Final analyses were conducted on 137 participants. We tested the difference in demographic and health-related variables between participants with CES-D scores less than or greater than/equal to 16 as shown in Table 1 and Table 2, respectively. In general, most participants were female (77.4%) and White (77.4%), and more than half (59.1%) had a CES-D score greater than 16, indicative of depressive symptomology. Significant variations were observed in CES-D score by sex (*p* = 0.009), race as a binary variable (*p* = 0.018), participants’ perceived feelings of safety at home (*p* = 0.008), stress (*p* < 0.001), body fat percentage (*p* = 0.005), LBM (*p* = 0.030), BMR (*p* = 0.013), and systolic blood pressure (*p* = 0.038). There were notable trends in education level (*p* = 0.062), cause of stress (*p* = 0.063), and self-reported chronic condition (*p* = 0.053). When assessing demographic and health factors by the IPAQ score threshold of 600 MET min/week, no differences were seen. 

### 3.2. Correlations between CES-D Scores and Health Factors

There was not a significant relationship observed between the CES-D score and IPAQ score (r = −0.106, *p* = 0.219). Analysis of body composition measures revealed that CES-D score was positively correlated with body fat percentage (r = 0.281, *p* < 0.001) and negatively correlated with LBM (r = −0.170, *p* = 0.047) as shown in Table 3. Conversely, the IPAQ score was positively correlated with LBM (r = 0.175, *p* = 0.041) and negatively correlated with body fat percentage (r = −0.285, *p* < 0.001). IPAQ score demonstrated a negative correlation with resting heart rate (r = −0.185, *p* = 0.031). However, its associations with mean arterial pressure (MAP), systolic, and diastolic blood pressure did not yield significant results. Resting heart rate displayed a significant positive correlation with body fat percentage (r = 0.228, *p* = 0.007) and a negative correlation with LBM (r = −0.231, *p* = 0.007). Notably, stress was also positively correlated with body fat percentage (r = 0.304, *p* = 0.001) and negatively correlated with LBM (r = −0.171, *p* = 0.046). 

The correlation analysis of IPAQ score intensities and domains is summarized in Table 4. A negative correlation was observed between CES-D score and MET min/week of MPA (r = −0.224, *p* = 0.008). Analysis of IPAQ score domains revealed they were not significantly related to CES-D score. Body fat percentage had an inverse relationship with MET min/week of VPA (r = −0.317, *p* < 0.001). Heart rate also demonstrated an inverse relationship with MET min/week of MPA (r = −0.180, *p* = 0.035). Comparably, LBM was positively associated with VPA (r = 0.221, *p* = 0.010). Stress was found to be inversely related to MET min/week in work (r = −0.181, *p* = 0.035) and leisure (r = −0.195, *p* = 0.022). 

### 3.3. Multivariate Models Assessing the Relationships between Depressive Symptoms and Physical Activity

The results of linear regressions are summarized in Table 5. Model 1 analyzed the total IPAQ score as a continuous variable, adjusting for sex, race, education level, body fat percentage, stress, and being a psychology major. Models 2 and 3 assessed the IPAQ score as categorical (0–600 MET min/week, 600–1499 MET min/week, and ≥1500) and as a binary variable (0–599 MET min/week and ≥600), respectively, while also controlling for sex, race, education level, body fat percentage, stress, and being a psychology major. When measured continuously and categorically, IPAQ was not a significant predictor of depressive symptoms. However, using it as a binary variable with a cutoff of 600 MET min/week narrowed the range of IPAQ scores and revealed a significant negative relationship with CES-D score (*β* = −0.169, SE= 2.748, *p* = 0.034). 

The IPAQ intensity and domain scores did not significantly contribute to the linear regression models; therefore, data were not included. The range of MET minutes per week might have been too broad to detect an effect. When used in logistic regression with CES-D as a binary variable, the IPAQ scores were not significant. Among the variables adjusted for, stress was significant across all models of linear and logistic regression. These findings emphasize the role of stress in influencing depressive symptoms. 

## 4. Discussion

The aim of this study was to explore the association between depressive symptoms, physical activity, and modifiable lifestyle factors among Hispanic emerging adults. Our results revealed a high prevalence of depressive symptoms in this population, with more than half of the participants exhibiting clinically significant symptomology. This is consistent with previous research revealing a heightened burden of mental health disorders in this demographic. Moreover, our study highlighted the complex interplay between physical activity, body composition, and depressive symptoms. Body fat percentage was positively correlated with CES-D score and negatively correlated with IPAQ score while LBM had the opposite relationship. Although results vary by sex, age, and other factors, depression has been associated with increased body fat percentage and decreased LBM in previous research from the Hispanic Community Health Study/Study of Latinos (HCHS/SOL), National Health and Nutrition Examination Survey and other studies [56,57,58,59,60]. More than 60% of participants in our study had body fat percentages exceeding 25%, and these individuals presented significantly higher CES-D scores. Similarly, those with more than 25% body fat had significantly lower IPAQ scores. These results emphasize the importance of considering body composition in future investigations aimed at understanding the relationship between modifiable lifestyle behaviors and depression.

Stress was also positively correlated with CES-D score and body fat percentage, and negatively correlated with LBM. Analysis of IPAQ score intensities and domains found that stress was inversely correlated with vigorous physical activity and activity performed during work and leisure time. Notably, stress consistently emerged as a predictor across all models of linear and logistic regression, highlighting its influence on mental health outcomes. Hispanics face adverse childhood experiences and significant stressors throughout the immigration and acculturation process [36,60,61,62]. Young adults experience increased stress levels due to academic pressure, financial concerns, and social expectations in the transition to adulthood. These stressors compound the risk of depression and altered body composition, emphasizing the need for interventions targeting stress management in this population. Future research should identify and employ culturally tailored stress reduction techniques to improve modifiable behaviors and depressive symptoms. 

Heart rate also emerged as a significant indicator in our assessment of the relationship between physical activity and depressive symptoms. Our findings revealed resting heart rate was negatively associated with IPAQ score and LBM, while it was positively correlated with body fat percentage. While heart rate also demonstrated an inverse relationship with IPAQ score intensities, the correlation only reached significance for MPA, suggesting that engaging in moderate-intensity physical activity may have pronounced effects on cardiovascular health. This aligns with well-established research indicating regular physical activity lowers resting heart rate [63]. 

Our study found an inverse relationship between depressive symptoms and physical activity when it was assessed dichotomously. Our findings suggest an IPAQ score of 600 MET min/week, indicating an adequate level of physical activity as recommended by the World Health Organization and U.S. Physical Activity Guidelines may confer protective effects against depressive symptoms among Hispanic emerging adults [12,13]. Additionally, our study sought to examine the role of sociodemographic factors in the relationship between depressive symptoms and physical activity. Several sociodemographic variables such as country of origin, time spent in the U.S., and health insurance status were analyzed due to their known impact on depression and mental health outcomes [53,54,55]. The associations observed between depressive symptoms and factors such as race and feelings of safety underscore the importance of considering sociodemographic factors in understanding mental health outcomes. Given that the majority of participants were recruited primarily from a university setting, reported residing in the U.S. for more than five years, and had health insurance, they may constitute a unique subgroup within the Hispanic population. Consequently, many of the factors hypothesized to impact depressive symptoms did not yield significant results. 

These findings have important implications for addressing mental health disparities among Hispanic emerging adults and reducing the risk of future multimorbidity in this population. The strengths of this study include its comprehensive collection and examination of data about modifiable lifestyle behaviors and depressive symptoms from a substantial sample of Hispanic emerging adults. Data collection included sociodemographic information, self-reported chronic conditions, and modifiable lifestyle behaviors such as physical activity and stress. Furthermore, by utilizing validated assessments such as the CES-D and IPAQ, several noteworthy associations were uncovered that contribute to the body of literature about physical activity and mental health trends in this population that has been understudied in this context. Our findings can be used to develop interventions and guidelines aimed at promoting physical activity and improving mental health outcomes in Hispanic emerging adults. 

Our study also has several limitations. While the IPAQ score provides information on the intensities of physical activity, it may not provide a full understanding of the context or quality of physical activity, such as the type of activities performed or the level of enjoyment experienced. Similarly, the IPAQ score does not distinguish between activities that promote social interaction or nature-based activities which have been shown to provide mental health benefits [7,64]. Measuring activity by intensity may not capture the health benefits unique to different exercise modalities. These factors may influence the effectiveness of physical activity in reducing depressive symptoms but are lacking in our analyses and current research. Furthermore, the cross-sectional design limits our ability to establish causality of depressive symptoms and offers only a snapshot into multiple modifiable lifestyle behaviors of participants that may be involved in mental health. Our sample size may have been too small to capture the effect of activity and other factors on depressive symptoms and ensure generalizability. Similarly, our sample consisted of predominantly White and female participants recruited from a university setting. To improve the generalizability of findings across diverse Hispanic subgroups, future studies should aim to recruit larger and more diverse samples. Additionally, the use of self-report measures could affect the accuracy of physical activity levels and introduce recall bias.

Future studies could leverage digital platforms and mobile health devices to measure physical activity more accurately and deliver interventions aimed at reducing depressive symptoms. These tools can provide real-time support, track progress and heart rate, and differentiate between exercise modalities [65,66,67]. Community and group-based physical activity interventions have been shown to increase activity in adolescents and young adults [68,69]. They also simultaneously provide social support and improve mental health outcomes [4,5]. Future research should explore how several sociocultural and modifiable factors may interact to mediate depressive symptoms. Understanding these interactions can help design more culturally tailored interventions that address the unique challenges faced by Hispanic emerging adults. 

## 5. Conclusions

In conclusion, our study sheds light on the relationship between physical activity and depressive symptoms among Hispanic emerging adults. Alarmingly, more than 60% of participants demonstrated a CES-D score greater than 16, indicative of depressive symptomology, and a body fat percentage greater than 25. Correlation analysis of body composition measures indicated that CES-D score had a positive relationship with body fat percentage and an inverse relationship with lean body mass. Conversely, IPAQ score was positively related to lean body mass and inversely related to body fat percentage. However, during regression analyses adjusting for body fat percentage, it was not significant. When measured continuously and categorically, IPAQ score was not a significant predictor of depressive symptoms. However, when used as a binary variable with a cutoff of 600 MET min/week, a significant negative relationship with CES-D score was revealed. Stress emerged as a predictor of depressive symptoms and was also positively correlated with body fat percentage and inversely correlated with lean body mass. 

Our findings suggest body composition measures may be more indicative of obesity and mental health risk during this age period. Future interventions should go beyond merely promoting physical activity and incorporate strategies to optimize modifiable lifestyle factors and reduce stress. Additionally, future research could explore the context and quality of physical activity to provide a better understanding of the effectiveness of various physical activity modalities in reducing depressive symptoms. It is also important to consider sociodemographic factors and cultural context in understanding mental health outcomes among this population. Our findings highlight the potential interplay between physical activity, body composition, stress, and health factors on depressive symptoms in Hispanic emerging adults. 

## Figures and Tables

**Table 1:** Demographic health characteristics by CES-D score (categorical results).

Characteristic	CES-D < 16 *n* (%)	CES-D ≥ 16 *n* (%)	Total *n* (%)	*p*-Value
Sex				0.009
Male	19 (13.9)	12 (8.8)	31 (22.6)	
Female	37 (27)	69 (50.4)	106 (77.4)	
Age				0.927
18–21	35 (25.5)	50 (36.5)	85 (62)	
22–25	21 (15.3)	31 (22.7)	52 (38)	
Race				0.018
White	49 (35.8)	57 (41.6)	106 (77.4)	
Non-White	7 (5.1)	24 (17.5)	31 (22.6)	
Time in the U.S.				0.852
≤5 years	9 (6.6)	14 (10.2)	23 (16.8)	
>5 years	47 (34.3)	67 (48.9)	114 (83.2)	
Primary Language				0.590 ^b^
English	41 (29.9)	62 (45.3)	103 (75.2)	
Spanish	14 (10.2)	19 (13.9)	33 (24.1)	
Portuguese	1 (0.7)	0 (0)	1 (0.7)	
Country of Origin				0.097
U.S.	28 (20.4)	52 (38)	80 (58.4)	
Outside of the U.S.	28 (20.4)	29 (21.2)	57 (41.6)	
Highest Level of Education				0.062 ^b^
H.S., Some college	30 (21.9)	39 (28.5)	69 (50.4)	
Associate degree	11 (8)	31 (22.6)	42 (30.7)	
Bachelor’s degree	12 (8.8)	9 (6.6)	21 (15.3)	
Master’s degree or greater	3 (2.2)	2 (1.5)	5 (3.6)	
Major				0.906 ^b^
Health	18 (13.1)	31 (22.6)	49 (35.8)	
STEM	20 (14.6)	28 (20.4)	48 (35)	
Finance	6 (4.4)	7 (5.1)	13 (9.5)	
PR/Marketing	4 (2.9)	4 (2.9)	8 (5.8)	
IR/Law/Criminology	4 (2.9)	7 (5.1)	11 (8)	
Education	2 (1.5)	3 (2.2)	5 (3.6)	
Non-degree seeking	1 (0.7)	0 (0)	1 (0.7)	
Not a student	1 (0.7)	0 (0)	1 (0.7)	
More than 1	0 (0)	1 (0.7)	1 (0.7)	
Dietetics Major				0.535
Yes	9 (6.6)	10 (7.3)	19 (13.9)	
No	47 (34.3)	71 (51.8)	118 (86.1)	
Psychology Major				0.148
Yes	7 (5.1)	18 (13.1)	25 (18.2)	
No	49 (35.8)	63 (46)	112 (81.8)	
Employment				0.575 ^b^
Full time	4 (2.9)	3 (2.2)	7 (5.1)	
Part time	24 (17.5)	35 (25.5)	59 (43.1)	
Unemployed	1 (0.7)	5 (3.6)	6 (4.4)	
Student	27 (19.7)	38 (27.7)	65 (47.4)	
Monthly Household Income				0.292
USD 0–1999	25 (18.4)	33 (24.3)	58 (42.6)	
USD 2000–4999	13 (9.6)	28 (20.6)	41 (30.1)	
USD 5000 or more	18 (13.2)	19 (14)	37 (27.2)	
Household Size				0.147
1–2	19 (13.9)	22 (16.1)	41 (29.9)	
3–4	34 (24.8)	46 (33.6)	80 (58.4)	
5 or more	3 (2.2)	13 (9.5)	16 (11.7)	
Feeling of Safety at Home				0.004 ^b^
Yes	52 (38)	58 (42.3)	110 (80.3)	
Maybe	4 (2.9)	20 (14.6)	24 (17.5)	
No	0 (0)	3 (2.2)	3 (2.2)	
Health Insurance Status				1.000 ^b^
Insured	52 (38)	75 (54.7)	127 (92.7)	
Uninsured	4 (2.9)	6 (4.4)	10 (7.3)	
Stress				<0.001 ^b^
Not at all	4 (2.9)	0 (0)	4 (2.9)	
A bit	15 (10.9)	6 (4.4)	21 (15.3)	
Something	9 (6.6)	18 (13.1)	27 (19.7)	
Enough	21 (15.3)	29 (21.2)	50 (36.5)	
A lot	5 (3.6)	28 (20.4)	33 (24.1)	
Stress Cause				0.063 ^b^
Finances	12 (8.8)	16 (11.7)	28 (20.4)	
Familial	4 (2.9)	17 (12.4)	21 (15.3)	
Work-related	22 (16.1)	17 (12.4)	39 (28.5)	
Health	2 (1.5)	7 (5.1)	9 (6.6)	
Pain	1 (0.7)	0 (0)	1 (0.7)	
Politics/Worldly Events	2 (1.5)	2 (1.5)	4 (3)	
Other	13 (9.5)	22 (16.1)	35 (25.5)	
Chronic Condition				0.053
Yes	19 (13.9)	41 (29.9)	60 (43.8)	
No	37 (27)	40 (29.2)	77 (56.2)	
Body Mass Index (kg/m^2^)				0.792 ^b^
Underweight < 18.5	2 (1.5)	4 (2.9)	6 (4.4)	
Healthy 18.5–25	30 (21.9)	36 (26.3)	66 (48.2)	
Overweight 25–30	14 (10.2)	24 (17.5)	38 (27.7)	
Obesity > 30	10 (7.3)	17 (12.4)	27 (19.7)	
Body Fat Percentage				0.030
7.6–25%	26 (19)	23 (16.8)	49 (35.8)	
25–53%	30 (21.9)	58 (42.3)	88 (64.2)	
IPAQ Categorically				0.616 ^b^
<600	6 (4.4)	13 (9.5)	19 (13.9)	
600–1499	6 (4.4)	6 (4.4)	12 (8.8)	
>1500	44 (32.1)	62 (45.3)	106 (77.4)	
IPAQ Binary				0.456
<600	6 (4.4)	13 (9.5)	19 (13.9)	
600 or more	50 (36.5)	68 (49.6)	118 (86.1)	

*p*-value obtained using Pearson Chi-square. ^b^ Indicates *p*-value from Fisher’s Exact Test.

**Table 2:** Health characteristics by CES-D score (continuous results).

Characteristic	CES-D < 16 Mean ± SD	CES-D ≥ 16 Mean ± SD	*p*-Value
IPAQ score (MET min/week)	4485.55 ± 3921.57	4734.30 ± 4897.51	0.752
MET min/week VPA	1824.14 ± 1970.25	1861.33 ± 2814.90	0.932
MET min/week MPA	1465.98 ± 1821.53	1173.06 ± 1914.41	0.371
MET min/week walking	1195.43 ± 1533.63	1699.91 ± 1723.10	0.081
Sitting time (min/week)	2137.68 ± 1262.5	2148.15 ± 1362.36	0.964
Weight (kg)	71.41 ± 15.38	70.57 ± 19.12	0.784
Lean body mass (kg)	50.92 ± 13.39	46.25 ± 11.41	0.030
Body fat %	26.67 ± 10.40	31.82 ± 10.31	0.005
BMI (kg/m^2^)	25.25 ± 4.27	26.37 ± 6.16	0.242
Basal metabolic rate (kcal)	1491.68 ± 262.23	1385.2 ± 229.88	0.013
Waist circumference (cm)	76.06 ± 10.90	77.512 ± 13.68	0.509
Hip circumference (cm)	98.25 ± 9.01	99.64 ± 11.38	0.446
Systolic blood pressure (mm Hg)	124.11 ± 13.19	119.72 ± 11.20	0.038
Diastolic blood pressure (mm Hg)	74.39 ± 8.50	74.00 ± 9.91	0.782
Heart rate (beats per minute)	79.64 ± 11.62	80.36 ± 14.14	0.755
Mean arterial pressure (mm Hg)	84.89 ± 8.58	84.51 ± 9.04	0.802

*p*-value obtained using independent-sample *t*-test.

**Table 3:** Correlations between CES-D or IPAQ score and health factors.

Health Factors	CES-D Score	IPAQ Score
Weight (kg)	r = −0.040	r = −0.064
Waist Circum (cm)	r = 0.029	r = −0.080
LBM (kg)	r = −0.170 *	r = 0.175 *
Body Fat %	r = 0.281 **	r = −0.285 **
Heart Rate (bpm)	r = 0.117	r = −0.185 *
MAP (mm Hg)	r = 0.069	r = −0.030
Stress	r = 0.432 **	r = −0.099

* Correlation is significant at the 0.05 level. ** Correlation is significant at the 0.01 level.

**Table 4:** Correlations between CES-D with IPAQ scores, sitting time, and IPAQ score intensities and domains.

IPAQ Scores	CES-D Score	Body Fat %	LBM (kg)	Heart Rate (bpm)	Stress
IPAQ Score	r = −0.106	r = −0.285 **	r = 0.175 *	r = −0.185 *	r = −0.099
Sitting Time	r = 0.081	r = 0.023	r = −0.054	r = 0.095	r = 0.081
MET min/week walking	r = 0.128	r = −0.029	r = −0.029	r = −0.102	r = 0.121
MET min/week MPA	r = −0.224 *	r = −0.102	r = 0.033	r = −0.180 *	r = −0.067
MET min/week VPA	r = −0.155	r = −0.317 **	r = 0.221 **	r = −0.136	r = −0.195 *
MET min/week work	r = −0.035	r = −0.025	r = 0.168 *	r = 0.022	r = −0.181 *
MET min/week transport	r = −0.004	r = −0.036	r = 0.158	r = 0.122	r = −0.136
MET min/week housework	r = −0.038	r = −0.021	r = 0.118	r = 0.094	r = −0.146
MET min/week leisure	r = −0.053	r = −0.053	r = 0.089	r = 0.113	r = −0.195 *

* Correlation is significant at the 0.05 level. ** Correlation is significant at the 0.01 level.

**Table 5:** Linear regression between CES-D score, IPAQ scores, and health factors.

	Predictor Variables		Model 1		Model 2		Model 3	
	β (SE)	*p* Value	β (SE)	*p* Value	β (SE)	*p* Value	β (SE)	*p* Value
Variable								
Sex	0.044 (2.558)	0.618	0.043 (2.570)	0.628	0.057 (2.578)	0.521	0.074 (2.554)	0.406
Race (binary)	−0.107 (2.235)	0.167	−0.107 (2.244)	0.171	−0.125 (2.277)	0.116	−0.134 (2.233)	0.085
Education Level	−0.090 (1.102)	0.249	−0.094 (1.130)	0.238	−0.085 (1.102)	0.273	−0.093 (1.087)	0.228
Body Fat %	0.136 (0.104)	0.137	0.143 (0.108)	0.132	0.111 (0.107)	0.236	0.102 (0.104)	0.266
Stress	0.354 (0.880)	<0.001	0.354 (0.883)	<0.001	0.362 (0.882)	<0.001	0.370 (0.872)	<0.001
Psych Major (y/n)	0.137 (2.439)	0.080	0.138 (2.452)	0.079	0.129 (2.448)	0.102	0.126 (2.412)	0.105
IPAQ Scores								
IPAQ Score			0.022 (0.001)	0.782				
IPAQ Category Score					−0.090 (1.373)	0.267		
IPAQ Binary Score							−0.169 (2.748)	0.034

Predicted confounding variables: sex, race, education level, body fat percentage, stress, and psychology major (y/n). Model 1 includes baseline covariates and IPAQ score. Model 2 includes baseline covariates and IPAQ Category Score. Model 3 includes baseline covariates and IPAQ Binary Score.

## Data Availability

Dataset available on request from the authors.
